# Probenecid Treatment Inhibits Replication of the Edmonston Measles Virus Strain in Vero Cells

**DOI:** 10.3390/v17111475

**Published:** 2025-11-05

**Authors:** Jackelyn Murray, David E. Martin, Ralph A. Tripp

**Affiliations:** 1 Department of Infectious Diseases, University of Georgia, Athens, GA 30605, USA; 2 TrippBio, Inc., Jacksonville, FL 32256, USA; davidmartin@trippbio.com

**Keywords:** measles virus, Edmonston strain, probenecid, viral replication

## Abstract

There are no FDA-approved antiviral treatments for measles virus (MeV). Management is mainly supportive care. MeV treatments may include vitamin A, ribavirin, the MeV vaccine, or human immunoglobulin for pregnant patients exposed to MeV but lacking immunity. The Edmonston strain of MeV serves as the basis for the MeV vaccine and remains a component of the measles-mumps-rubella (MMR) vaccine. We previously showed that probenecid can be used therapeutically to prevent the replication of several key respiratory viruses. This study indicates that pre-treatment with probenecid (prophylaxis) can inhibit the replication of the Edmonston MeV strain in VeroE6 cells (1.12 μM) and Vero-SLAM cells (1.03 μM), while treatment (1 h post-infection, hpi) inhibits replication in VeroE6 cells (1.32 μM) and Vero-SLAM cells (8.66 μM). These results suggest that probenecid is an effective, host-directed antiviral drug against MeV replication in vitro.

## 1. Introduction

MeV is an enveloped virus with a single-stranded, negative-sense RNA genome and belongs to the genus Morbillivirus within the family Paramyxoviridae. MeV is a disease primarily affecting children and spreads through the air via droplets and aerosols, which can remain airborne for up to two hours. MeV is highly contagious, with a basic reproductive number (Ro) estimated to be between 12 and 18, meaning that, on average, one person infected with MeV can infect 12 to 18 other susceptible individuals [1]. An individual with MeV is contagious for four days before developing a rash and remains contagious for four days after it appears. Vaccinated individuals who contract MeV often experience milder symptoms.

MeV was declared eliminated in the United States in 2000, following decades of high vaccination coverage that reduced annual cases from hundreds of thousands to fewer than 100 per year. However, the past decade has seen periodic resurgences, with notable spikes in 2014 (667 cases), 2019 (1274 cases), and now a dramatic surge in 2025. As of 7 October 2025, more than 1563 confirmed cases have been reported across 42 states. This is the highest annual total since 2000 and a more than 5-fold increase over 2024. Most 2025 cases have occurred within large outbreaks, primarily affecting the under-vaccinated communities, with 92% of cases in unvaccinated or unknown-status individuals. Up to 12% of patients have required hospitalization, and 3 deaths have been reported, all among unvaccinated persons. This resurgence occurs amid a nationwide decline in the rate of MMR vaccination below the 95% threshold required for herd immunity and transmission in close-contact, under-vaccinated groups.

MeV infection can cause a phenomenon known as “immune amnesia”, which erases immune memory, making individuals susceptible to other infections for months or even years. MeV targets specific immune cells, including memory B cells and invariant T cells, leading to their destruction [2]. However, immunity from a previous MeV infection or receiving two doses of the MMR vaccine provides long-lasting protection; nonetheless, it is possible to become susceptible again if someone becomes immunocompromised [3].

Current MeV treatments may include therapy with vitamin A, ribavirin, the MMR vaccine, or human immunoglobulin. Vitamin A is administered to address vitamin A deficiency related to MeV. This treatment helps prevent severe complications such as eye damage, blindness, and death associated with MeV [4], but it does not prevent viral replication. Ribavirin is a broad-spectrum antiviral used to treat respiratory infections, as demonstrated by its in vitro activity against MeV [5]. Although ribavirin has been used for severe MeV cases or in immunocompromised patients, strong clinical efficacy data are lacking. Crucially, the trough plasma concentrations of ribavirin in humans with standard dosing (2–7 μM) are generally below the reported IC50 values for MeV inhibition in vitro, which range from approximately 3–50 μM [6]. These pharmacokinetic and antiviral potency data suggest that ribavirin alone is unlikely to have significant antiviral activity in humans due to insufficient systemic exposure relative to the concentrations needed to suppress MeV replication. Ribavirin is approved for certain paramyxovirus infections, excluding MeV, and is used alongside IFNα therapies. It has been clinically tested against MeV, primarily for treating patients with Subacute Sclerosing Panencephalitis (SSPE). This rare, progressive neurodegenerative disease arises as a complication of MeV infection [7]. High-dose ribavirin, either alone or combined with IFNα, appears to be more effective against MeV than IFNα alone [8]. This paucity of effective antiviral treatment options leaves vaccination as the only reliable therapeutic approach, but it is not without limitations. Specifically, vaccination does not serve as a treatment after exposure. Even if vaccination begins during an MeV outbreak, it takes weeks to develop robust immunity, underscoring the importance of effective therapies to bridge the vaccination gap.

The first live, attenuated vaccine, known as Edmonston B, was licensed in the United States and was eventually replaced by the more attenuated Moraten strain [9]. The Edmonston strain of MeV has been the only MeV vaccine used in the U.S. since 1968. Wild-type MeV is commonly isolated from B95a cells, a marmoset lymphoblastoid cell line, and shows restricted host cell specificity, replicating minimally in Vero cells. However, Edmonston MeV, a lab strain, reproduces in various cell types, including Vero cells [10]. MeV spreads in lymphoid organs throughout the body and produces syncytia by utilizing the signaling lymphocyte activation molecule (SLAM) as a receptor [11]. It may also spread in SLAM-negative epithelial tissues. CD46 is another receptor for vaccine strains of MeV but not for wild-type MeV. Therefore, we examined whether Edmonston MeV could replicate following probenecid treatment of VeroE6 or Vero-SLAM cells in vitro, as there are currently no antivirals available that inhibit MeV replication.

In this study, we evaluated the prophylactic and therapeutic effects of probenecid on VeroE6 and Vero-SLAM cells infected (MOI = 0.1) with Edmonston MeV. The rationale was that probenecid is safe, non-cytotoxic, and has been used in pharmacological treatments that affect both endogenous substances, such as uric acid, and exogenous substances, such as penicillin [12]. It has also been shown to modulate the activity of various membrane channels and transport proteins. Additionally, probenecid exhibits potent antiviral activity in the picomolar to nanomolar range against a broad spectrum of respiratory viruses, including influenza strains, SARS-CoV-2 variants, human metapneumovirus (HMPV), respiratory syncytial virus (RSV), and others. As a host-directed antiviral, probenecid influences both antiviral and anti-inflammatory responses. Recent studies have demonstrated its anti-inflammatory effects on the NLRP3 inflammasome and MAPK signaling pathways, affecting several components of the MAPK cascade [13,14,15]. A strategy combining prophylactic (vaccination) and therapeutic (antiviral) approaches could help overcome obstacles in controlling MeV; however, this approach requires identifying antivirals that are both safe and effective against MeV. The results show that pre-treatment with probenecid (prophylaxis) can inhibit the replication of the Edmonston MeV strain in VeroE6 cells (1.12 μM) and Vero-SLAM cells (1.03 μM). Treatment administered 1 hpi also inhibits replication in VeroE6 cells (1.32 μM) and Vero-SLAM cells (8.66 μM).

## 2. Materials and Methods

### 2.1. Cell Culture

VeroE6 cells (CCL-81) from the American Type Culture Collection (ATCC, Manassas, VA, USA) were cultured in Dulbecco’s Modified Eagle Medium (DMEM; ThermoFisher, Waltham, MA, USA). Vero-SLAM cells (generously provided by Melinda Brindley, University of Georgia) were propagated in the presence of G418 (400 µg/mL, Sigma, St. Louis, MO, Canada) for selection. The cells were kept in the log phase at 37 °C in a 5% CO_2_ incubator and used for all studies. All cell lines were immediately frozen upon receipt, kept at low passage numbers, and tested PCR-negative for mycoplasma contamination (ThermoFisher, Waltham, MA, USA).

### 2.2. Viruses

MeV strain Edmonston (BEI Resources, ATCC) was propagated in VeroE6 cells. A viral stock was prepared by infecting Vero cells at a multiplicity of infection (MOI = 0.1) and incubating for 72 h at 37 °C with 5% CO_2_. Virus titer was quantified using a plaque assay with Vero cells.

### 2.3. Probenecid

Probenecid (Sigma, St. Louis, MO, USA; CAS Number: 57-66-9) was diluted in DMSO (Sigma) and resuspended in DMEM supplemented with 2% FBS (Cytivia, Logan, UT, USA), which was warmed to 37 °C, to achieve the desired concentrations (e.g., 100 µM = 100 µL of 10 mM stock in 10 mL media). DMSO has a range of cytotoxicity to cell concentrations ranging from 0.5 to 40% *v*/*v*, with toxicity occurring at 1% and higher for most cell types. The exact threshold varies depending on the specific cell line, incubation time, and other factors. DMSO was used as a control. All materials were kept at 37 °C throughout to prevent the precipitation of probenecid.

### 2.4. MeV Plaque Assay

Tenfold serial dilutions of infected cell supernatants were added to confluent Vero cell monolayers in a 6-well plate (Corning, Corning, NY, USA). After virus adsorption for 1 h at 37 °C with 5% CO_2_, the cell monolayers were overlaid with DMEM containing 2% methylcellulose (Sigma) and incubated at 37 °C for 5 days. To count plaques, the overlay was removed, and the monolayers were fixed with a 60:40 acetone:methanol (Sigma) solution for 20 min at room temperature. Cells were then counterstained with crystal violet (Sigma), and plaques were enumerated.

### 2.5. Probenecid Treatment

Probenecid (Sigma) dilutions were prepared from a 100 mM working stock. For prophylactic or therapeutic treatment studies, VeroE6 or Vero-SLAM cells were pretreated prophylactically for 24 h before infection or therapeutically treated at 1 hpi with probenecid at various concentrations, i.e., 1000 μM, 500 μM, 250 μM, 100 μM, 50 μM, 25 μM, 12 μM, 6 μM, 3 μM, 1 μM, 0.5 μM, and 0 μM. The cells were infected at an MOI of 0.01. After virus adsorption for 1 h at 37 °C and 5% CO_2_, the cell monolayers were overlaid with infection media containing different concentrations of probenecid. The virus was incubated for 48 h at 37 °C and 5% CO_2_. After 48 h, the supernatants were removed, and the plates were tested by plaque assays using crystal violet (Sigma) as a counterstain. Our lab has tested the cytotoxicity of probenecid on various mammalian cells, including A549, Vero E6, Calu-3, Hep-2, and NHBE cells. These studies involved treating cells with probenecid and then measuring cell viability using an Alamar Blue assay (ThermoFisher, Waltham, MA, USA) [16]. Briefly, cells were treated with probenecid, and Alamar Blue was added. The plate was incubated, and the cells were read using a microplate reader. The number of viable cells was determined by measuring the absorbance of the dye. This assay was performed multiple times under various assay and cell conditions to confirm that the antiviral activity was specific to probenecid. Several published studies that involved this analysis are noted [17,18,19,20,21,22].

### 2.6. Statistics

GraphPad Prism 9 (GraphPad Software, Boston, MA, USA) was used to calculate the IC50 values using a nonlinear regression model based on the Hill equation.

## 3. Results

**Figure 1:**
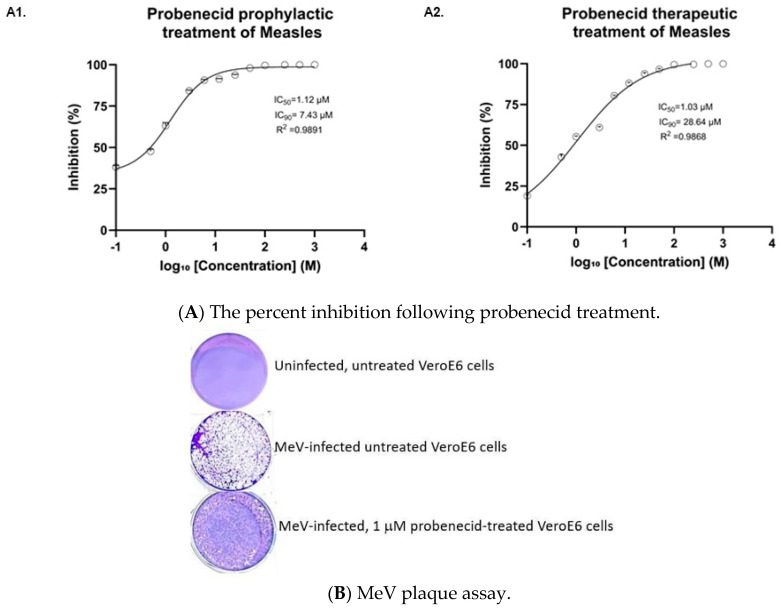
(**A**) VeroE6 cells were (**A1**) prophylactically treated with probenecid 24 h before infection with Edmonston MeV (MOI = 0.1), or (**A2**) treated 1 h after infection with probenecid at the specified concentrations. After 48 h, cell lysates were added to VeroE6 cells for plaque assay analysis. Statistical analysis was performed via a nonlinear regression model based on the Hill equation. (**B**) is a representative well from a 6-well MeV plaque assay of VeroE6 cells from uninfected, MeV-infected, or MeV-infected 1 µM probenecid-treated VeroE6 cells. The purple-colored cell lawn shows crystal violet staining. The holes in the cell lawn reflect MeV plaques. The efficacy of 1 µM probenecid treatment is indicated by the vastly reduced MeV plaques in the cell lawn.

**Figure 2:**
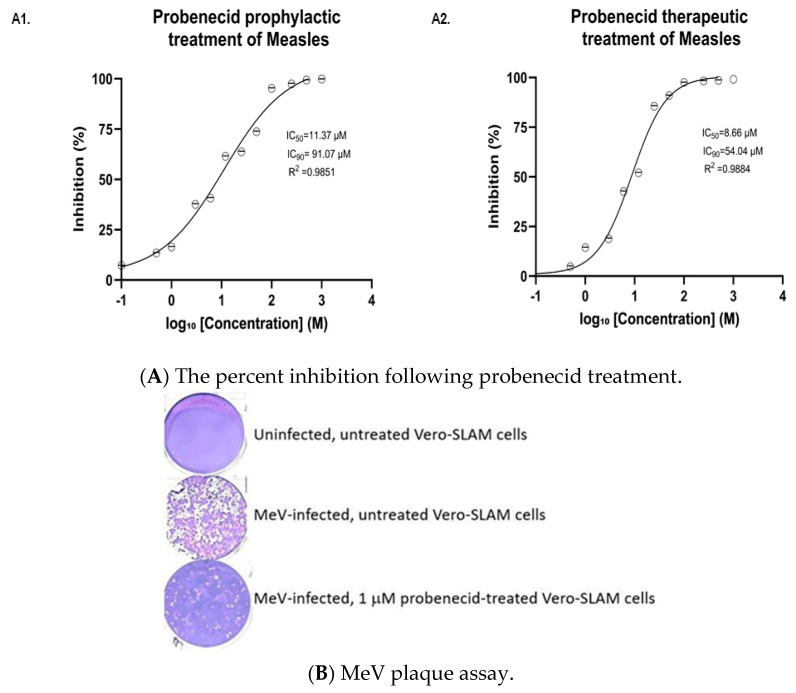
(**A**) Vero-SLAM cells were (**A1**) prophylactically treated with probenecid 24 h before infection with Edmonston MeV (MOI = 0.1), or (**A2**) treated 1 h after infection with probenecid at the specified concentrations. After 48 h, cell lysates were added to Vero-SLAM cells for plaque assay analysis. Statistical analysis was performed via a nonlinear regression model based on the Hill equation. (**B**) is a representative well from a 6-well MeV plaque assay of Vero-SLAM cells from uninfected, MeV-infected, or MeV-infected 1 µM probenecid-treated Vero-SLAM cells. The purple-colored cell lawn shows crystal violet staining. The holes in the cell lawn reflect MeV plaques. The efficacy of 1 µM probenecid treatment is indicated by the vastly reduced MeV plaques in the cell lawn.

This study examined the efficacy of probenecid treatment before and after MeV infection (MOI = 0.1) of VeroE6 and Vero-SLAM at 24, 48, and 72 hpi. The 24 hpi time point did not show measurable virus plaques; however, peak detectable virus plaque numbers in both VeroE6 and Vero-SLAM cells occurred at 48 hpi (Figure 1 and Figure 2). High plaque numbers were evident at 72 hpi; however, the plaques were not countable because MeV replication in untreated VeroE6 and Vero-SLAM cells destroyed the cell lawns. The values shown below represent the results of 3 independent experiments and 3 replicas/experiment. The study described below examined VeroE6 cells infected (MOI = 0.1) with Edmonston MeV and treated with probenecid. Effective IC50 values for VeroE6 cells that were (A1) prophylactically treated with probenecid 24 h before infection with Edmonston MeV (MOI of 0.1), or (A2) treated 1 h after infection with probenecid, were obtained at the concentrations shown in Figure 1A.

The study below examined Vero-SLAM cells infected (MOI = 0.1) with Edmonston MeV and treated with probenecid (Figure 2A). IC50 values for Vero-SLAM cells were measured under two conditions: (A1) prophylactically treated with probenecid 24 h before infection with Edmonston MeV (MOI = 0.1), or (A2) treated 1 h after infection at various concentrations, as shown in Figure 2A.

## 4. Discussion

Probenecid is non-cytotoxic and does not affect the virus [12]. Probenecid is a host-directed drug that inhibits the virus replication machinery in the host cell. Recent studies examining the mechanism of action of probenecid on viral replication shows that treatment inhibits mitogen-activated protein kinase (MAPK) signaling pathways, particularly the JNK and ERK pathways, which are crucial for virus replication and inflammation [13,14,15], as well as directly blocking the function of the OAT3 transporter [22]. Additionally, probenecid disrupts the formation of the activator protein-1 (AP-1) transcription complex, which has a crucial role in regulating gene expression in response to various cellular signals, including inflammation [23]. The activity of probenecid on host cell pathways, rather than directly on the virus, makes it a host-directed therapy with broad applicability against multiple viral strains that use the same conserved MAPK pathways for viral replication [24].

Humans are the natural reservoir for MeV; however, MeV can infect nonhuman primates, with the transmission cycle being difficult to sustain in wild populations [25]. Other morbilliviruses in the same family, such as canine distemper virus (CDV), can infect a broader range of mammals; however, there is no animal model other than non-human primates that recapitulates the hallmarks of human measles. Nevertheless, the CDV-ferret model provides a viable system for de-risking drugs before formal development [26]. One small molecule inhibitor, specifically ERDRP-0519, was first identified in a high-throughput screen and blocks the polymerase activity of all currently circulating MeV genotypes. ERDRP-0519 is a small-molecule antiviral designed to inhibit the activity of morbilliviruses by targeting the morbillivirus RNA-dependent RNA polymerase complex. It is highly effective against lethal infection of ferrets with CDV, a surrogate model for human MeV [27]. It has been shown that prophylactic and post-exposure treatments prevent measles disease in squirrel monkeys [28]. Initiating therapy at the onset of clinical signs can reduce virus shedding, potentially helping to control outbreaks. Results suggest that the drug could alleviate MeV symptoms and aid in MeV eradication. In contrast, the anti-MeV inhibitor ERDRP-0519 faces challenges in advancing beyond Phase I safety trials in adult volunteers as it must meet strict safety standards suitable for pediatric patients. Conversely, probenecid is a safe and effective medication approved by the FDA. The pediatric dose, used as an adjunct to antibiotic therapy for children aged 2 to 14 years weighing less than 50 kg, is 25 mg/kg orally once or 40 mg/kg/day divided into four doses.

Challenges in identifying suitable animal models have impeded the development of MeV therapies. One limitation is that most small animal models for MeV focus on viral infection rather than replication. Probenecid does not block infection but instead blocks replication; thus, a cotton rat or transgenic mouse model that expresses the human MeV receptor SLAM will not effectively evaluate probenecid’s efficacy [29]. Cotton rats are naturally susceptible and may aid in the study of viral pathogenesis and immune suppression. Transgenic models, especially those that are also immunocompromised (e.g., lacking interferon response), can better mimic aspects of human infection, including disease symptoms and immunosuppression [30]. However, they remain unsuitable for assessing the inhibition of viral replication mediated by probenecid. The key question is whether the host pathways employed by MeV during replication are sufficiently similar to those in humans, since probenecid’s effectiveness may not be replicated, and NHPs are either too costly or prohibited for use.

In summary, the results of this study confirm the broad-spectrum antiviral activity of probenecid and support its continued evaluation as an oral therapeutic for MeV infection. Due to the ongoing public health crisis, these data represent an initial assessment of the antiviral activity of probenecid. Future studies will expand the breadth of activity to include other MeV isolates and relevant animal studies.

## Data Availability

The data are stored at the Tripp Lab at UGA and are freely available.
